# Comprehensive study into the activation of the plasma enzyme systems during attacks of hereditary angioedema due to C1-inhibitor deficiency

**DOI:** 10.1186/s13023-015-0351-5

**Published:** 2015-10-09

**Authors:** Dorottya Csuka, Nóra Veszeli, Éva Imreh, Zsuzsanna Zotter, Judit Skopál, Zoltán Prohászka, Lilian Varga, Henriette Farkas

**Affiliations:** 3rd Department of Internal Medicine, Semmelweis University, Faculty of Medicine, Kútvölgyi út 4, H-1125 Budapest, Hungary; Department of Cardiology, Heart & Vascular Center, Semmelweis University, Budapest, Hungary

**Keywords:** Hereditary angioedema, C1-inhibitor, Edematous attack, Plasma enzyme systems, Attack location

## Abstract

**Background:**

The activation of plasma enzyme systems contributes to hereditary angioedema attacks. We aimed to study the activation markers of the fibrinolytic, coagulation, and contact systems in a larger number of paired samples obtained from the same C1-INH-HAE patients in symptom-free periods and during attacks.

**Methods:**

Eleven parameters (Factors XI, XII, and C1-inhibitor activity; the concentrations of the D-dimer, prothrombin fragments 1 + 2, plasminogen, plasminogen activator inhibitor-1 [PAI-1], thrombin-anti-thrombin III [TAT] complex, fibrinogen) were measured along with prothrombin time and activated partial thromboplastin time (aPTT), using commercial kits. We compared these markers in samples obtained from the same 39 patients during attack-free periods and during 62 edematous episodes. Forty healthy subjects of matching sex and age served as controls.

**Results:**

Compared with the healthy controls, significantly higher FXI and FXII activity (*p* = 0.0007, *p* = 0.005), as well as D-dimer (*p* < 0.0001), prothrombin fragments 1 + 2 (*p* < 0.0001), and TAT (*p* = 0.0303) levels were ascertained in the patients during symptom-free periods. The evaluation of samples from symptom-free periods or obtained during attacks revealed the increase of FXII activity, as well as of the concentration of D-dimer, prothrombin fragments 1 + 2, and TAT during edematous episodes. PAI-1 level, prothrombin time, and aPTT decreased significantly during attacks, compared with symptom-free periods. D-dimer level was significantly higher during multiple- *vs*. single-site attacks.

**Conclusions:**

Comparing a large number of paired samples from symptom-free periods or from edematous episodes allowed accurate appraisal of the changes occurring during attacks. Moreover, our study pointed out that individual episodes may be characterized by different marker patterns.

## Background

C1-inhibitor (C1-INH) – the deficiency of which causes hereditary angioedema (C1-INH-HAE) types I and II – is a regulator of the complement, contact, coagulation, and fibrinolytic systems, as it inhibits rapidly activated factor XII (FXIIa), activated factor XI (FXIa), and kallikrein [1–5]. The contact system, also known as the plasma kallikrein-kinin system, consists of factor XII, prekallikrein, high-molecular-weight kininogen (HK), and factor XI. Factor XII can be activated by contact with negatively charged surfaces, extracellular RNA [6], misfolded proteins [7], inorganic polyphosphates released from bacteria [8, 9], and bacterial surfaces [10].

When proteins of the contact system bind to endothelial cells, the activation of factor XII leads to the conversion of prekallikrein to kallikrein [11], which in turn reciprocally activates factor XII. Furthermore, physical trauma and surgery (known trigger factors of C1-INH-HAE) [12] can activate the contact system via the stress-induced release of HSP-90, or the direct endothelial cell activation of circulating factor XII, during which large areas of endothelium are exposed [13]. Moreover, these stimuli are important activators of the coagulation cascade through the expression of tissue factor and the activation of factor VII [14]. Activated factor XII can initiate blood coagulation via factor XI that links the extrinsic and intrinsic arms of the coagulation system. Factor XI circulates bound to high-molecular-weight kininogen in the plasma, and it is activated by FXIIa. FXIa in turn can activate factor XI, thereby contributing to the formation of a fibrin clot, and also to thrombin generation via the intrinsic pathway of coagulation. Remarkably, thrombin may increase vascular permeability by the cleavage and activation of a thrombin-susceptible receptor on the endothelial cell surface. Binding induces intercellular gap formation [15], which leads to edema [14, 16, 17].

Once activated by factor XII, kallikrein cleaves its enzyme cofactor, high-molecular-weight kininogen, leading to the release of the vasoactive substance, bradykinin – the key mediator of edematous attacks. The binding of bradykinin to the bradykinin B2 receptor on the surface of endothelial cells activates several intracellular signaling pathways that induce vasodilatation, as well as increase vascular permeability and fluid efflux [18, 19]. Furthermore, bradykinin can stimulate the release of tissue plasminogen activator in the human vasculature [20]; it also suppresses PAI-1 gene expression [21].

Additionally, kallikrein activates the fibrinolytic system either directly – by converting plasminogen to plasmin [22–24], or indirectly – by activating tissue-type (tPA) and urokinase-type (uPA) plasmin activators, which convert plasminogen to plasmin. Interestingly, plasmin can activate factor XII to factor XIIa as an alternative to the kallikrein feedback [25]. In vitro studies showed that plasmin enhances the production of bradykinin through the action of kallikrein on high-molecular-weight kininogen [26].

Previous studies have analyzed the changes that occur during C1-INH-HAE attacks in each plasma enzyme system separately. According to their findings activation of the plasma enzyme systems occurs during attacks as suggested by the elevated levels of cleaved HK, FXIIa, activated factor VII [14, 27, 28], prothrombin fragments 1 + 2 (a marker of thrombin generation), and D-dimer (a marker of fibrin degradation) [14, 29]. Despite all these facts, thrombotic complications during the attacks, or increased thrombotic risk in patients with C1-INH-HAE are not characteristic, even though the activation of the contact system is poorly regulated. It is hypothesized that in patients with C1-INH-HAE, the activation of factor XII preferentially triggers prekallikrein activation, rather than the production of FXIa by FXIIa.

Although the studies mentioned in the foregoing were milestones in the exploration of the pathomechanism of C1-INH-HAE, they had their limitations. They enrolled only a small number of C1-INH-HAE patients, and usually did not compare the symptom-free and during-attack states of the same patients. Furthermore, measuring the components of the plasma enzyme systems separately has the disadvantage that the results of the assays do neither reflect the true balance between the activators and inhibitors in the original blood sample, nor the interaction of the four plasma enzyme systems. In our study, we tried to overcome these limitations by the simultaneous evaluation of the changes occurring in the coagulation, fibrinolytic, and kinin-kalllikrein systems during C1-INH-HAE episodes. Furthermore, we compared this large set of parameters in the same patients during symptom-free periods and during attacks – that is, each patient served as his/her own control.

## Methods

### Study subjects

#### C1-INH-HAE patients

Thirty-nine C1-INH-HAE patients (12 men and 27 women, median age: 35 years, 25 to 75^th^ percentiles: 22–50 years), 33 with type I, and 6 with type II of C1-INH-HAE, were enrolled into our study. In each patient, the diagnosis was established according to the accepted clinical and laboratory criteria (positive family history, clinical symptoms of angioedema, low functional C1-INH level, low C4, normal C1q concentrations) [30]. Human plasma-derived C1-INH concentrate (pdC1-INH); Berinert®, CSL Behring, Marburg, Germany) was available as an acute remedy for edematous attacks. All patients received pdC1-INH concentrate whenever this was necessary to relieve the most severe forms of edematous episodes (i.e. upper airway, abdominal, facial, genital, and severe limb edema, predominantly). Seventeen patients (6 male and 11 female) received long-term prophylaxis during the study period; thirteen of them were on long-term prophylaxis with danazol, and four of them were on tranexamic acid. Twenty-two patients have not been on long-term prophylaxis.

The “symptom-free samples” were collected during the annual control visits, in case at least two weeks elapsed since the date of the last attack. Furthermore, 62 samples in total were collected from the patients during edematous attacks, before administering pdC1-INH concentrate (at least one, but not more than 5 “during-attack” samples were obtained per patient). The severity and the site of edematous attacks, as well as the time from onset to blood sampling were recorded in the Hungarian HAE Registry. The distribution of attack sites was as follows: 30.6 % (19/62) – abdominal region, 24.3 % (15/62) – arm, 19.3 % (12/62) – subcutaneous and submucosal locations simultaneously, 9.7 % (6/62) – leg, 8.1 % (5/62) – trunk, 4.8 % (3/62) – face, 3.2 % (2/62) – upper airways.

#### Healthy controls

The control group consisted of 40 healthy adults (15 men and 25 women, median age: 33 years, 25 to 75^th^ percentiles: 21–58 years). All subjects had been referred for routine medical evaluation, and volunteered for the study by giving informed consent. The healthy controls did not have any known disease, or receive medicinal products at the time of blood sampling. C1-INH deficiency was excluded by complement testing in all healthy subjects.

C1-INH-HAE patients and controls were not statistically different as regards age and gender distribution.

### Blood sampling

Serum and citrated plasma samples obtained from patients with C1-INH-HAE in symptom-free periods and during attacks were stored at −80 °C until processing. Peripheral blood samples were drawn also from the healthy subjects, as prescribed by the study protocol. The latter was approved by the institutional review board of Semmelweis University of Budapest, and informed consent was obtained from the participants in accordance with the Declaration of Helsinki.

### Measurement of the parameters related to the contact, fibrinolytic, coagulation, and complement systems

All analyzed parameters were determined using the same, unthawed aliquot from each subject and each assay was performed on aliquots thawed for the same time. Commercial kits were used to determine the activity of C1-INH in serum (Quidel, San Diego, USA), and in citrated plasma the coagulation markers (prothrombin fragments 1 + 2 and thrombin-antithrombin complex, Enzygnost), the proteins of the fibrinolytic system (plasminogen: Berichrom Plasminogen kit, and PAI-1: Berichrom), the activation marker of fibrinolysis (D-dimer, Innovance D-dimer kit), as well as the activity of factor XI (Siemens Healthcare Diagnostics) and factor XII (Siemens Healthcare Diagnostics), according to the manufacturer’s instructions. Prothrombin time and activated partial thromboplastin time (aPTT) were determined by standard laboratory methods in citrated plasma. The level of fibrinogen was determined using the Clauss method using citrated plasma, where high levels of thrombin are added to diluted plasma and the time it takes for that thrombin to convert fibrinogen to fibrin correlates to the concentration of fibrinogen [31].

### Statistical analysis

The statistical calculations were performed with Prism for Windows v5.02 (GraphPad Software Inc., San Diego, CA, www.graphpad.com), and SPSS v13.0 (SPSS Inc., Chicago, IL). Mann–Whitney’s U-test was used to compare two independent groups (C1-INH-HAE patients *vs.* healthy controls), whereas the Wilcoxon test was chosen to compare the “symptom-free” and “during-attack” results of the same patients. If a patient had several “during-attack samples”, a mean value was calculated for paired statistical comparison. In order to take into account all attack samples, a linear mixed model (LMM) was fitted to estimate the changes between the “symptom-free” and “during-attack” states of the same patients. This model permits including all the data obtained during various attacks of different patients (random effects), and even allows for missing data. In this model, the “symptom-free” or the “during-attack” (type of sample) was included in the LMM as a fixed effect, whereas patient ID, and the number of successive attacks were included as random effects to adjust for correlation among repeated measures within the subjects. The different markers of the contact, fibrinolytic, coagulation, and complement systems were each included as a dependent variable in the LMM.

When calculating correlations using Spearman’s rho, each patient was characterized by a random value for each parameter. All the statistical analyses were two-tailed, and *p* < 0.05 was considered to represent a significant difference, or correlation.

## Results

### Evaluation of coagulation parameters in symptom-free periods and during attacks, in the same C1-INH-HAE patients

In order to analyze the activation of the coagulation cascade during edematous attacks – as it had been suggested previously – we determined the plasma coagulation parameters in samples collected from the same C1-INH-HAE patients in symptom-free periods and during attacks. Then, we compared their results to the values of healthy subjects.

**Fig. 1:**
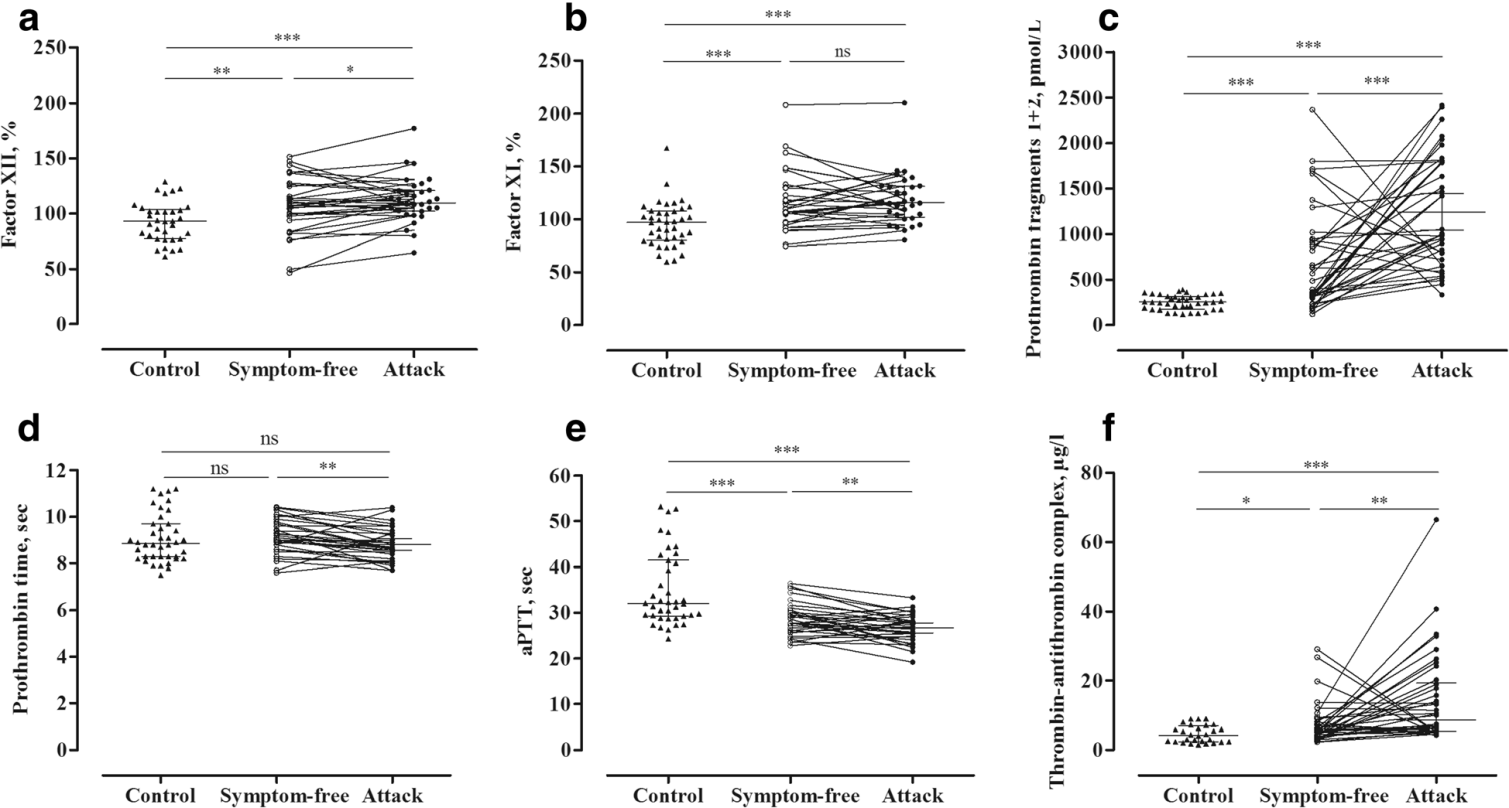
Evaluation of coagulation parameters in symptom-free periods and during attacks in the same C1-INH-HAE patients(Panels **a-f**). Each patient is represented by a single value, which was calculated as the mean in case several attacks had occurred. The results of the patients and those of the healthy controls were compared using the Mann–Whitney test; whereas the “symptom-free” or “during attack” results of the same patients were compared with the paired *t*-test

The activity of factor XII was enhanced in the symptom-free period, compared with that observed in the healthy subjects (*p* = 0.0050), and increased further significantly during attacks (*paired t-test, p* = 0.0314) in the same C1-INH-HAE patients (Fig. 1a).

Interestingly, the activity of factor XI was significantly elevated in C1-INH-HAE patients, both in the symptom-free period (*p* = 0.0007) and during attacks (*p* < 0.0001), compared with that seen in the healthy subjects. However, no further elevation was ascertained during attacks, in comparison to the symptom-free period (Fig. 1b).

The concentration of prothrombin fragments 1 + 2 was elevated in the symptom-free period compared with the healthy subjects (*p* < 0.0001), and increased significantly further in the same patients during attacks (*paired t-test, p* = 0.0005) (Fig. 1c).

Although we could not find a difference in prothrombin time between C1-INH-HAE patients and healthy subjects, prothrombin time was significantly shorter during attacks, compared with the symptom-free period (*paired t-test, p* = 0.0026) (Fig. 1d).

Interestingly, activated partial thromboplastin time was shorter, both in the symptom-free period (*p* = 0.0001) and during attacks (*p* < 0.0001), compared with the healthy controls. A further decrease was observed during attacks, compared with the symptom-free period (*paired t-test, p* = 0.0096) (Fig. 1e).

The level of thrombin-antithrombin complexes was higher both in symptom-free (*p* = 0.0303) and in symptomatic (*p* < 0.0001) C1-INH-HAE patients, compared with the controls. We detected a stepwise elevation in its level from the symptom-free period to the attacks (*paired t-test, p* = 0.0028) (Fig. 1f).

### Analysis of the markers and proteins of fibrinolysis in symptom-free periods and during attacks in the same C1-INH-HAE patients

**Fig. 2:**
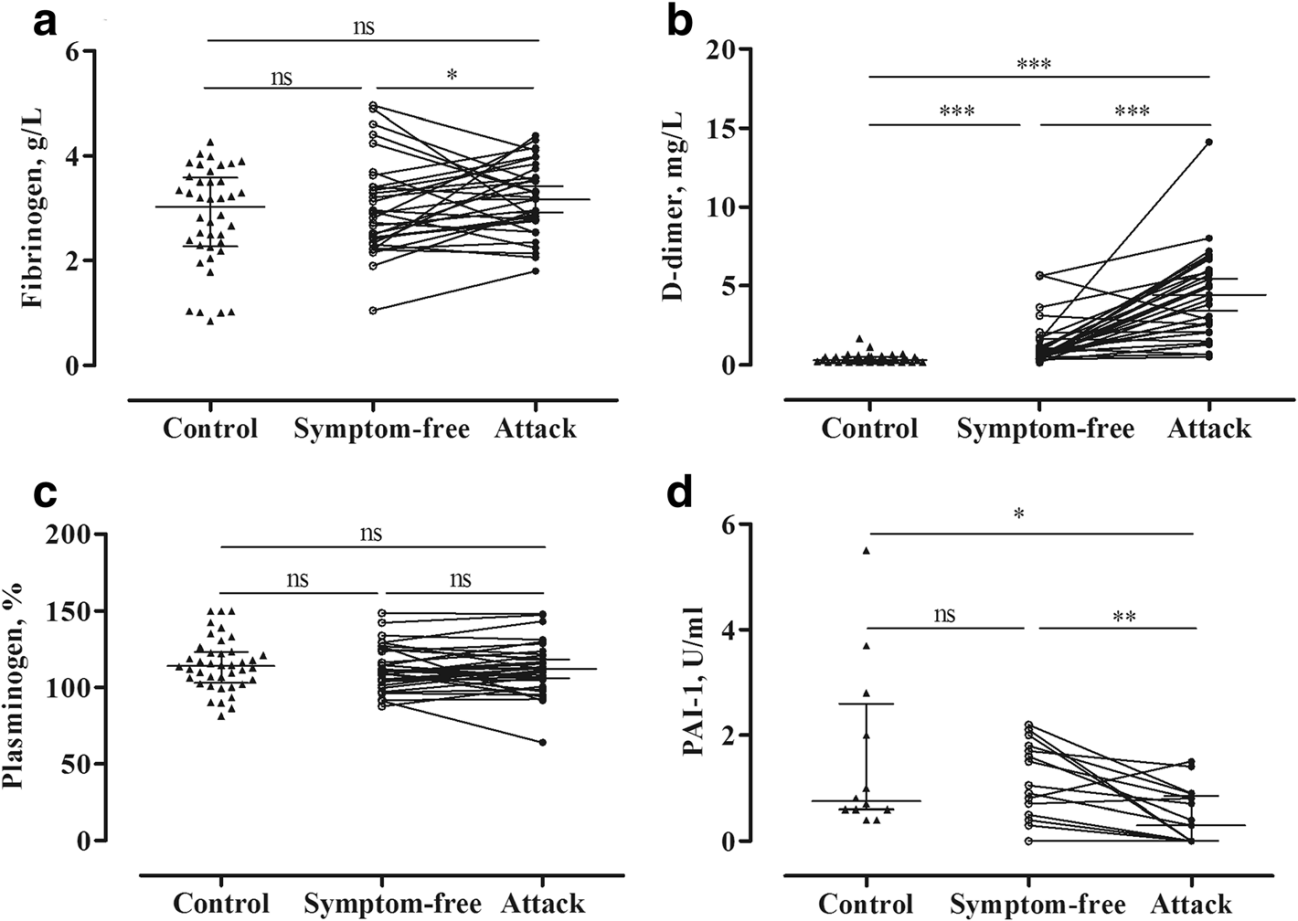
Analysis of the proteins and markers of fibrinolysis in symptom-free periods and during attacks in the same C1-INH-HAE patients (Panels **a-d**). Each patient is represented by a single value that was calculated as the mean in case several attacks had occurred. The results of the patients and of the healthy controls were compared using the Mann–Whitney test; whereas the “symptom-free” or “during attack” results of the same patients were compared with the paired t-test. PAI-1 levels were available only from 12 healthy subjects

The concentration of fibrinogen was slightly elevated during attacks, compared with the symptom-free period (*p* = 0.012) (Fig. 2a), but no significant difference was found between healthy subjects and patients during the symptom-free period. D-dimer level showed a significant elevation both in the symptom-free period (*p* < 0.0001) and during attacks (*p* < 0.0001), compared with the healthy subjects. Furthermore, its level increased further during attacks (*paired t-test, p* < 0.0001), compared with the symptom-free period of the same patients (Fig. 2b).

By contrast, the level of plasminogen activator inhibitor-1 (PAI-1) was lower during attacks than in the symptom-free period (*paired t-test, p* = 0.0040), or in the healthy subjects (*p* = 0.0213) (Fig. 2d).

We could not find any difference between the study groups as regards plasminogen level (Fig. 2c).

We applied a mixed linear model to analyze the changes of the parameters of the fibrinolytic, coagulation, and kinin-kallikrein systems during attacks, in comparison with reference samples (obtained in an attack-free period). This model permits including all the data obtained during successive attacks of different patients (random effects), and even allows for missing data. We found the corresponding values from the symptom-free and during-attack states (fixed effect) to be significantly different, which confirms the results of the paired t-test performed beforehand (Table 1).

**Table 1:** The median levels of the proteins and markers of plasma enzyme systems in symptom-free periods and during attacks in the same C1-INH-HAE patients. The Linear Mixed Model was used on all measured parameters to simulate random effects and repeated measures, as well as to detect differences between the “symptom-free” and the “during-attack” states of the same patients

Dependent variable	Estimate^a^	95 % Confidence interval		*P*-value^a^
		Lower limit	Upper limit	
Factor XII, %	5.657	5.644	5.671	0.019
Factor XI, %	4.365	4.350	4.380	0.118
Prothrombin fragments 1 + 2, pmol/L	387.251	386.257	388.245	0.001
Prothrombin time, sec	−0.533	−0.534	−0.534	<0.0001
Activated partial thromboplastin time, sec	−3.039	−3.041	−3.037	<0.0001
Thrombin-anti-thrombin complex, μg/L	4.981	4.971	4.991	0.004
Fibrinogen, g/L	0.414	0.414	0.414	0.012
D-dimer, mg/L	3.105	3.104	3.106	<0.0001
Plasminogen, %	1.731	1.721	1.741	0.361
Plasminogen activator inhibitor-1, U/ml	−0.524	−0.525	−0.525	0.006

^a^For the difference between the “during attack” and the “symptom-free” samples

### Correlations among the study parameters of healthy controls and of C1-INH-HAE patients in the symptom-free period and during attacks

**Fig. 3:**
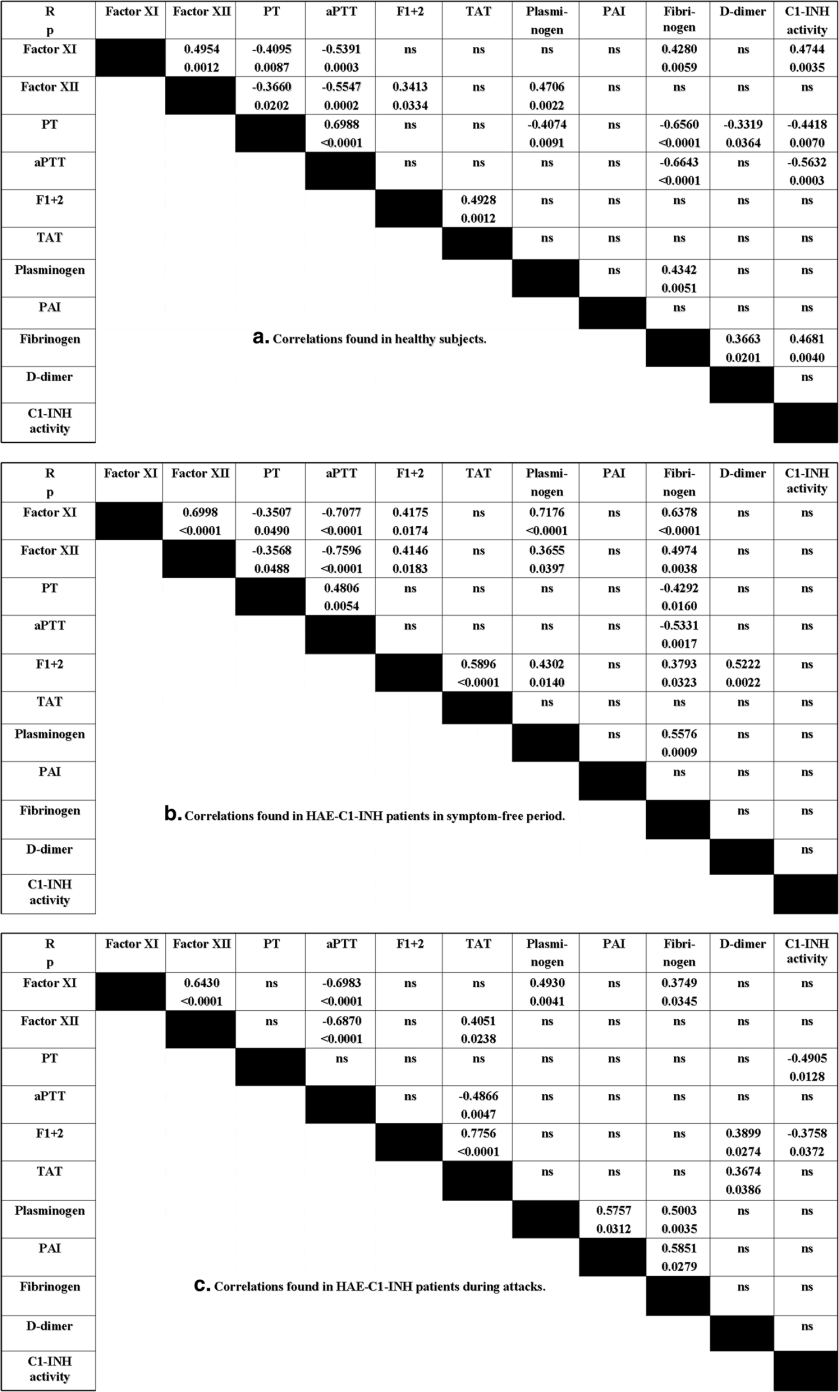
Correlations among the study parameters in healthy controls (**a**) and in C1-INH-HAE patients during the symptom-free period (**b**), and during attacks (**c**). Each patient is represented by one random value. Spearman’s rank correlation coefficient was calculated

Next, we evaluated the correlations between the study parameters. In healthy subjects, the closest correlations were found between aPTT and the activity of factor XI (*R* = −0.5391, *p* = 0.0003), the activity of factor XII (*R* = −0.5547, *p* = 0.0002), prothrombin time (*R* = 0.6988, *p* < 0.0001), the level of fibrinogen (*R* = −0.6643, *p* < 0.0001), and C1-INH activity (*R* = −0.5632, *p* = 0.0003). Furthermore, the activity of C1-INH correlated significantly with that of factor XI (*R* = 0.4744, *p* = 0.0035) and with fibrinogen level (*R* = 0.4681, *p* = 0.0040) (Fig. 3a).

In the analyses of the correlations identified in C1-INH-HAE patients, each patient was represented by a random value. In symptom-free patients, we found closer correlations, for instance between aPTT and factor XI (*R* = −0.7077, *p* < 0.0001), factor XII (*R* = −0.7596, *p* < 0.0001), or fibrinogen (*R* = −0.5331, *p* = 0.0017). Furthermore, fibrinogen level correlated significantly with the activity of factor XI (*R* = 0.6378, *p* < 0.0001) or of factor XII (*R* = 0.4974, *p* = 0.0038), as well as with prothrombin time (*R* = −0.4292, *p* = 0.0160). Significant, positive correlations were found between the level of prothrombin fragments 1 + 2 and the concentrations of TAT (*R* = 0.5896, *p* < 0.0001), or D-dimer (*R* = 0.5222, *p* = 0.0022) in the symptom-free period (Fig. 3b).

Interestingly, we found a different correlation pattern during attacks, compared with that seen in the symptom-free period. The relationship between the levels of prothrombin fragments 1 + 2 and those of TAT became stronger (*R* = 0.7756, *p* < 0.0001), whereas the correlation between aPTT and factor XI (*R* = −0.6983, *p* < 0.0001) or factor XII (*R* = −0.6870, *p* < 0.0001) remained nearly the same. In contrast to the symptom-free period, the activity of C1-INH showed significant negative correlations with prothrombin time (*R* = −0.4905, *p* = 0.0128), and with the level of prothrombin fragments 1 + 2 (*R* = −0.3758, *p* = 0.0372) during attacks. Furthermore, D-dimer level correlated significantly with the concentration of prothrombin fragments 1 + 2 (*R* = 0.3899, *p* = 0.0274), or of TAT (*R* = 0.3674, *p* = 0.0386) (Fig. 3c).

None of the parameters correlated significantly with time from symptom onset to blood sampling.

### Differences between the coagulation and the fibrinolysis parameters, depending on attack location

We analyzed whether there is a difference between “single-site” *vs.* “multiple-site” attacks, or between the subcutaneous *vs.* submucosal episodes, as regards the levels of the measured parameters.

**Fig. 4:**
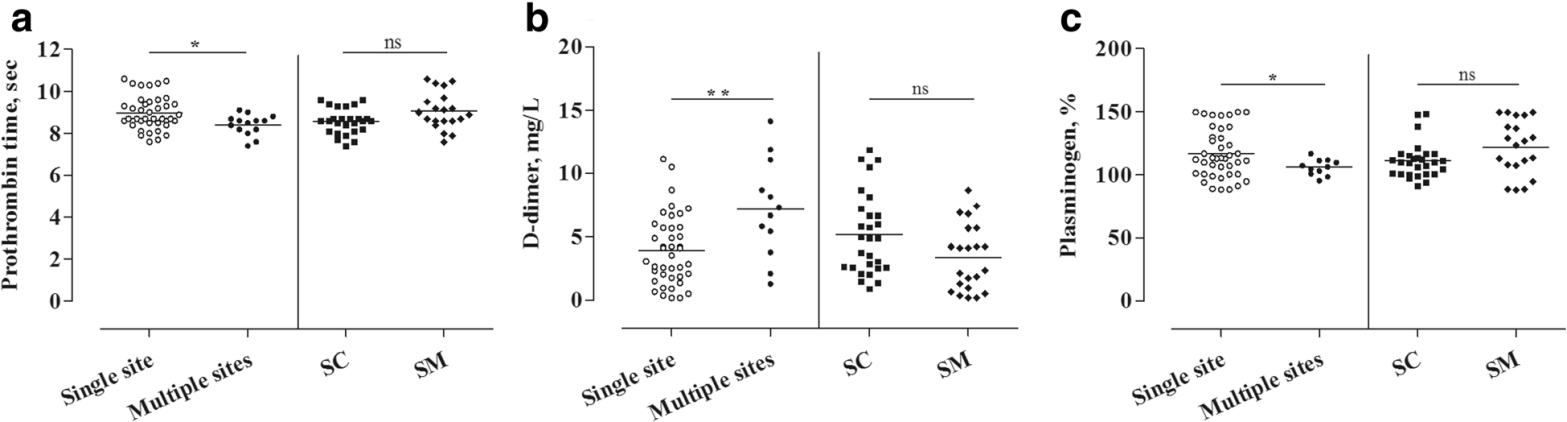
Differences in the coagulation and fibrinolysis parameters depending on attack location(Panels **a-c**). The LMM was used to compare the levels of the parameters during attacks occurring at a “Single site” vs. at “Multiple sites”, or in subcutaneous (SC) vs. submucosal (SM) location

Among all studied parameters, prothrombin time, D-dimer and plasminogen levels showed significant differences between attacks that had occurred in a single site vs. those involving multiple sites (Fig. 4).

During attacks occurring at multiple sites, the level of D-dimer increased (*p* = 0.002), whereas prothrombin time (*p* = 0.018) and plasminogen (*p* = 0.008) level decreased compared with the values of “single site” attacks (Table 2).

**Table 2:** Parameters of the coagulation and of the fibrinolytic system in symptom-free periods and during attacks, in the same C1-INH-HAE patients. The Linear Mixed Model was used to simulate random effects and repeated measures, to detect differences between the attacks occurring at a “Single site” *vs*. at “Multiple sites”, or in subcutaneous (SC) *vs*. submucosal (SM) location

Dependent variable	Comparison made between:	Estimate^a^	95 % Confidence interval		*P* value^a^
			Lower bound	Upper bound	
Prothrombin time, sec	Attack at single site *vs.* multiple sites	−0.478	−0.590	−0.367	0.018
	Attack in SC or SM location	0.052	−0.004	0.109	0.816
D-dimer, mg/L	Attack at single site *vs.* multiple sites	3.504	2.699	4.308	0.002
	Attack in SC or SM location	−1.737	−1.953	−1.522	0.060
Plasminogen, %	Attack at single site *vs.* multiple sites	−16.619	−21.280	−11.960	0.008
	Attack in SC or SM location	10.778	8.763	12.792	0.051

^a^For the difference between the “during attack” and the “symptom-free” samples

Furthermore, the concentration of D-dimer decreased slightly during submucosal attacks, whereas the level of plasminogen increased a little during submucosal compared with subcutaneous attacks. However, these differences were only of marginal significance (*p* = 0.060; *p* = 0.051, respectively) (Table 2).

All these data suggest that the activation of the plasma enzyme systems is more pronounced during attacks involving multiple sites, compared with those occurring at a single site. We could not find similar differences between the subcutaneous or submucosal location.

### Coagulation and fibrinolysis parameters in successive during-attack samples from the same patients

**Fig. 5:**
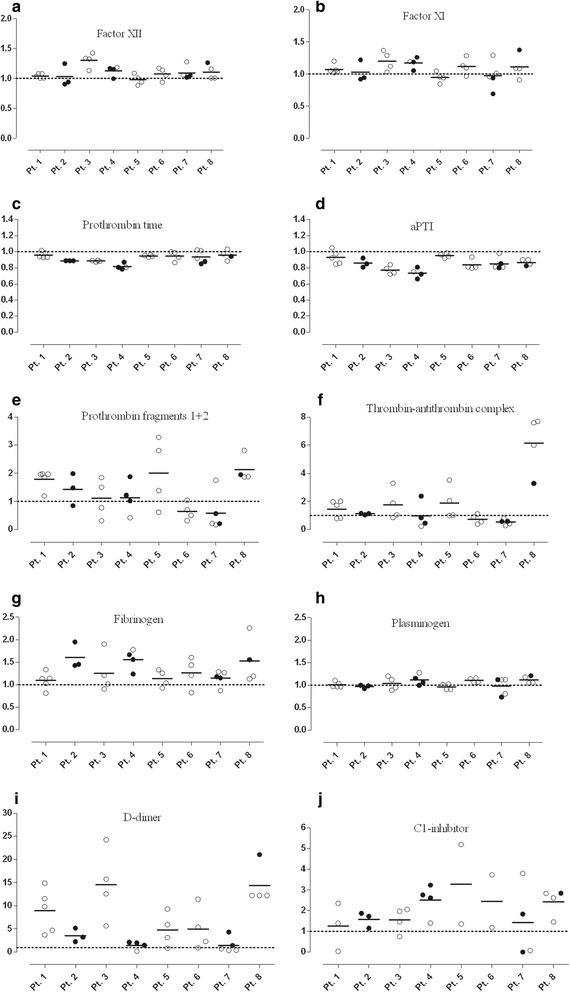
Distribution of the studied parameters during multiple attacks in the same patients with C1-INH-HAE. Factor XII (**a**), factor XI (**b**), prothrombin time (**c**), activated partial thromboplastin time (**d**), prothrombin fragments 1 + 2 (**e**), thrombin-antithrombin complex (**f**), fibrinogen (**g**), plasminogen (**h**), D-dimer (**i**) and C1-INH (**j**) were assessed in 8 patients with more than one “during attack” sample. Each “during attack” value was divided by the corresponding inter-attack value, and these ratios were plotted as different symbols, according to the site of the attack. The medians of the ratios are presented as a solid line. The ratio = 1 is presented as a dotted line. The values under the dotted line represent decreased levels, whereas those above the dotted line correspond to elevated levels of the measured parameters. Meaning of the symbols: empty symbols = single attack; filled symbols = multiple attacks

The severity and presentation of the manifestations of C1-INH-HAE, as well as its possible triggering factors exhibit considerable inter- and intra-individual variation. Therefore, we examined the magnitude of the changes and fluctuations in the study parameters during successive attacks experienced by the individual patients, as well as the range of their variability in a given subject. Eight patients (5 patients with C1-INH-HAE type I: Pts N° 1–5, and 3 patients with C1-INH-HAE type II: Pts N° 6–8) had ≥1 during-attack samples (min. 2 samples, max. 5 samples). This enabled us to monitor the changes even between successive attacks occurring in the same patient. Each “during-attack” value was divided by the corresponding “inter-attack” value, and these ratios were plotted as different symbols, according to the site of the attack (Fig. 5).

We found differences between the patients as regards the patterns of the coagulation and of the fibrinolysis parameters. In particular, the levels of fibrinogen, thrombin-antithrombin complex, D-dimer, and C1-inhibitor showed great variability among the study subjects (Fig. 5).

Furthermore, D-dimer level and the activity of the C1-inhibitor showed large fluctuations even between successive attacks in the same patient (Fig. 5).

## Discussion

**Fig. 6:**
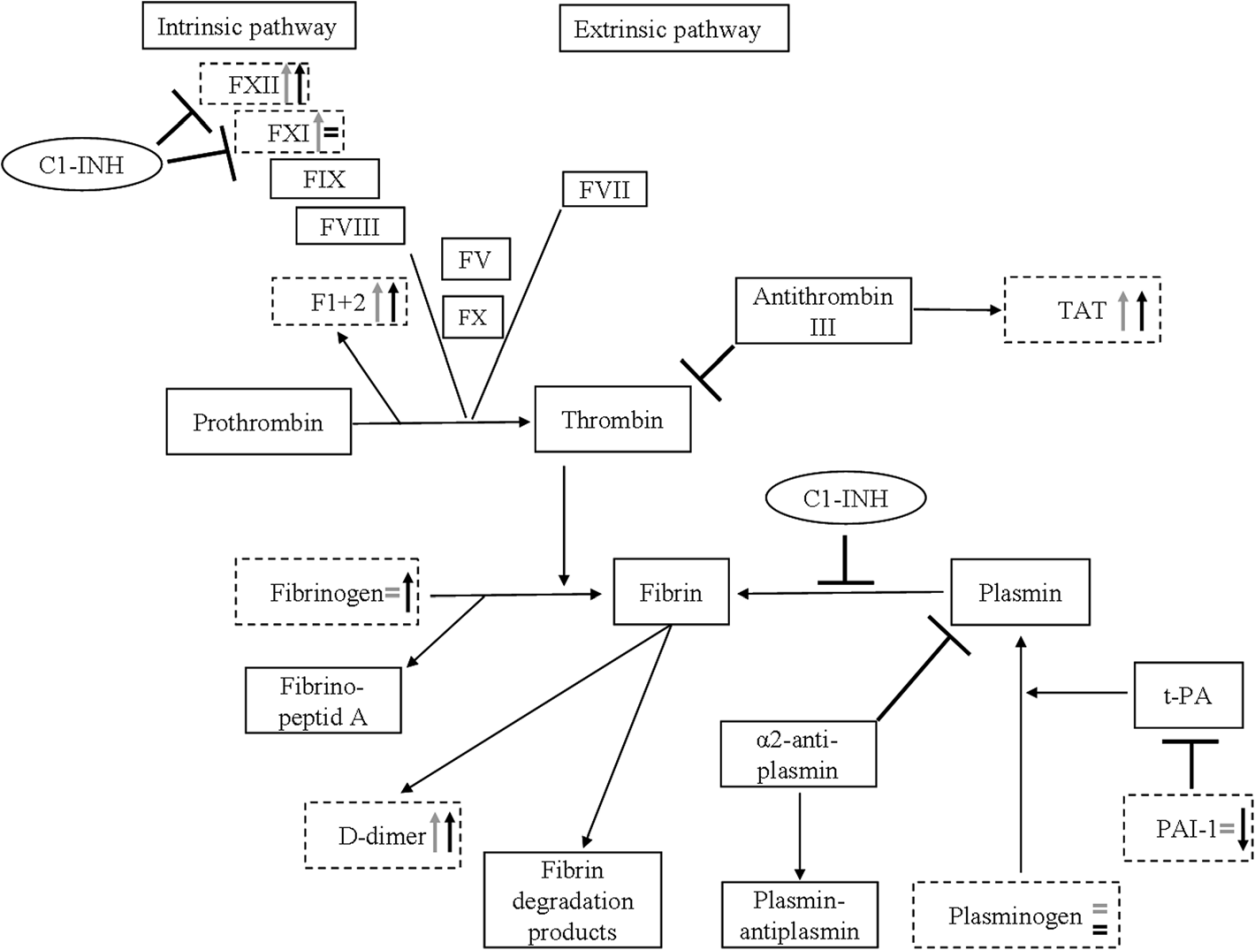
Schematic representation of the plasma enzyme systems, including a summary of our results. Grey arrows show the difference between healthy subjects and the symptom-free C1-INH-HAE patients, whereas black arrows show the difference between the symptom-free and the “during attack” period of the same patient. Dotted lines highlight those parameters that were analysed in our study. ⊣ The symbol indicates an inhibitory effect

Our aim was to perform a comprehensive study in a large population of C1-INH-HAE patients, in order to investigate the changes occurring in the plasma enzyme systems during edematous attacks. In the current study, we simultaneously evaluated such changes in the coagulation, the fibrinolytic, and the contact systems. Furthermore, we compared this large set of parameters recorded from the same patients in symptom-free periods and during attacks – that is, each patient served as his/her own control. The design of our study enabled us to conduct an investigation of unprecedented comprehensiveness and complexity into the changes accompanying C1-INH-HAE attacks. Figure 6 provides a comprehensive and schematic representation of the changes that occur in the plasma enzyme systems during edematous attacks.

In recent years, many studies have been published on the activation of the plasma enzyme systems during hereditary angioedema attacks. However, these included only a small number of patients and in most cases, the “symptom-free” and the “during attack” samples were obtained from different subjects.

Based on our results, the activity of factor XII is higher in the patients during the symptom-free period than in the healthy subjects and it increases further significantly during attacks; the latter is in accord with previous findings [14, 32].

Although a previous study did not detect any difference between healthy controls and C1-INH-HAE patients as regards the level of factor XI [33], we ascertained significant elevation of the latter in symptom-free C1-INH-HAE patients. However, we could not detect any further increase during attacks, compared with the symptom-free period.

In agreement with previous studies [29, 34], the level of prothrombin fragments 1 + 2 was higher in symptom-free C1-INH-HAE patients than in healthy controls, and it increased further in the same patients during attacks. Intriguingly, patients with C1-INH deficiency and with elevated levels of prothrombin fragments 1 + 2 (which indicate a state of hypercoagulability) do not have an increased risk for thrombotic events, as would be expected in such a situation. We had not measured the levels of coagulation inhibitors and thus, could not ascertain the magnitude and direction of their changes – or the possible development of a steady state of some kind. It is conceivable that the hyperfibrinolytic state, which we confirmed in our current study, and that was demonstrated in previous studies as the elevation of D-dimer levels – is sufficient to counteract the observed hypercoagulability [14, 33, 35]. D-dimer is produced during the breakdown of the fibrin mesh by plasmin. Therefore, in the state of hypercoagulation, any clot that forms is cleared by the fibrinolytic activity and thus, D-dimer is generated. In the context of our study, this may mean secondary hyperfibrinolysis, because the plasminogen levels measured during symptom-free periods did not differ from those determined in the healthy controls, or from those recorded during edematous attacks. If a major increase of hyperfibrinolysis would have occurred, we should have detected decreased plasminogen levels. Hypercoagulation, on the other hand, appeared prominent – because its presence was indicated even by the reduction of prothrombin time and of aPTT. It should be noted that the reduction of clotting times can be shown only occasionally, although this succeeds sometimes in disseminated intravascular coagulation [36]. In addition to the decreased clotting times, the elevated levels of the markers of the activation of coagulation were also characteristic in our study during the attacks. Of these, prothrombin fragments 1 + 2 indicates the formation of thrombin, whereas the elevation of TAT level reflects the neutralization of the formed thrombin by antithrombin. As thromboembolic events did not occur in our patients, it is possible that the thrombin thus formed is kept under control by the inhibitors.

The decrease of thrombin time indicates activation of the extrinsic pathway in the first place. However, judged by the activation of factors XII and XI, activation of the intrinsic pathway of blood coagulation is more typical. This is the possible reason why the reduction of aPTT is more conspicuous in symptom-free periods – and much more during attacks – even in comparison with healthy controls. To our best knowledge, activated partial thromboplastin time was not studied previously. In our study, we could detect a decreased level during the symptom-free period, compared to the healthy subjects, and its level decreased further during attacks.

One previous study did not find any difference between the TAT levels of healthy controls and of symptom-free patients [33]. Notwithstanding this, we detected a stepwise elevation in its level, both in the symptom-free period, and during the attacks. Thrombin generation appears to increase both in symptom-free/symptomatic C1-INH-HAE patients (as shown by the elevated level of the relevant plasma marker, prothrombin fragments 1 + 2). However, the effect of thrombin on vascular permeability may be weaker *in vivo*, owing to its rapid inactivation by anti-thrombin – as shown by the elevated levels of the TAT complex in our study. This inactivation may also explain why C1-INH-HAE patients do not develop thrombosis during edematous attacks.

To our best knowledge, the levels of fibrinogen and plasminogen have not yet been studied before in C1-INH-HAE patients. In our study, we could not find any difference in these parameters between healthy subjects and C1-INH-HAE patients. However, fibrinogen level was slightly elevated during attacks, compared with the symptom-free period – this may be explained by the fact that fibrinogen is an acute phase protein [37, 38]. Remarkably, PTT decreases during an acute phase reaction, just as we have seen in the C1-INH-HAE patients during attacks. Thus, the latter may be accompanied by an acute phase reaction.

In our study, D-dimer (the plasma marker of fibrin degradation) showed a significant elevation in the symptom-free period compared with the controls; this is in agreement with findings from previous studies [29, 34]. Interestingly, its level increased further during attacks of the same patients – as shown by a previous study as well [35]. At variance with a previous study, the level of PAI-1 decreased continuously in the symptom-free period and even further during attacks. However, no difference was found previously between the symptom-free and the symptomatic period [34]. As discussed by van Geffen et al., decreased levels of PAI-1 may be a consequence of enhanced thrombin production in association with protein C activation, which leads to the consumption of PAI-1 [34].

Taken together, our study confirmed the results of previous research into the activation of the coagulation system (as shown previously by elevated levels of prothrombin fragments 1 + 2) [33], as well as into the activation of the fibrinolytic system (as shown previously by the increased concentrations of D-dimer, TAFI, thrombomodulin and plasmin-α2-antiplasmin complex) [29, 34] in the symptom-free period.

Unlike the earlier studies [29, 33, 34], we detected differences in multiple parameters (factor XI, D-dimer, PAI-1) between the symptom-free and the during-attack states. The likely explanation for these is the small number of patients evaluated in the previous studies, of which only a few analyzed blood samples obtained from the same patients during the symptom-free period as well as during attacks.

The design of our study – that is, simultaneous evaluation of the components of the various plasma enzyme systems in the same patients – enabled us to investigate the correlations within and between the individual enzyme systems as far as the study parameters are concerned. We described for the first time that in C1-INH-HAE patients, the interactions among the plasma enzyme systems change both during symptom-free periods and during attacks, as suggested by the diverse correlation patterns, compared with the healthy subjects. The correlation between aPTT and factor XI or factor XII was present in all three sets of data (healthy subjects, symptom-free C1-INH-HAE, during attack C1-INH-HAE). Factor XII and aPTT exhibited a significant, negative correlation (which was the weakest in healthy subjects). This means that the higher is the level of factor XII, the shorter (smaller) is aPTT, and coagulation time decreases accordingly. However, this mechanism is possibly counterbalanced by the activation of the fibrinolytic system, as suggested by our results and by previous studies [14, 33, 35]. In healthy subjects, the C1-inhibitor – the key regulator in C1-INH-HAE – correlated with aPTT, the activity of factor XI, and with fibrinogen level. Interestingly, C1-INH activity showed no correlation in the symptom-free period, but during attacks, its activity exhibited a significant negative correlation with prothrombin time, or with the level of prothrombin fragments 1 + 2. The latter observation may indicate the involvement of the activation of the coagulation system during C1-INH-HAE attacks, in agreement with the findings of a previous study [34]. During attacks, the relationship between prothrombin fragments 1 + 2 and TAT became stronger than that observed in the symptom-free period. Despite this, the relationship between the levels of the D-dimer and of the prothrombin fragments 1 + 2 became weaker during attacks, compared with that seen in the symptom-free period. We can conclude that the strongest correlations were found in HAE-C1-INH patients during the symptom-free period.

The novelties of our study include the comparison of several plasma enzyme system components between attacks occurring at single *vs.* multiple sites, and in subcutaneous *vs.* submucosal locations. During attacks with multiple sites, we observed enhanced activation of the plasma enzyme systems, compared with attacks restricted to a single site. This was reflected by the significant elevation of D-dimer levels (that was also shown by Reshef et al.) [35], and by the decreased prothrombin time and plasminogen concentration in multiple-site attacks. Interestingly, we could not find any difference in the study parameters between subcutaneous and submucosal attacks. To our best knowledge, two studies have analyzed so far the possible differences between subcutaneous vs. submucosal attacks [29, 35], and only one of them showed significant difference in the level of D-dimer between these two locations [35].

As several during-attack samples were available from the same C1-INH-HAE patients, we analyzed whether there is a fluctuation between the attacks occurring in different patients, or between the successive attacks of the same patient. Interestingly, we found differences between the patients as regards the patterns of coagulation and fibrinolysis markers, namely in the levels of fibrinogen, thrombin-antithrombin complex, D-dimer and C1-inhibitor showed large inter-individual variations. Furthermore, D-dimer level and the activity of the C1-inhibitor exhibited a large fluctuation between the successive attacks of the same patient.

## Conclusions

The strengths of our study include the comparison of a large number of paired samples from symptom-free periods or from edematous episodes of the same patients, as well as the parallel monitoring of the parameters of the plasma enzyme systems. This approach allowed accurate appraisal of the changes occurring during C1-INH-HAE attacks. Moreover, our study pointed out that the individual edematous episodes may be characterized by different marker patterns among the studied individuals. We also showed that attacks with multiple sites are characterized by more enhanced activation of the plasma enzyme systems, compared with attacks restricted to a single site.

## Abbreviations

aPTT: Activated partial thromboplastin time
C1-INH: C1-inhibitor
C1-INH-HAE: Hereditary angioedema due to the deficiency of C1-INH
FXIa: Activated factor XI
FXIIa: Activated factor XII
LMM: Linear mixed model
PAI-1: Plasminogen activator inhibitor-1
TAT: Thrombin-antithrombin complex
tPA: Tissue-type plasmin activator
uPA: Urokinase-type plasmin activator
